# The Effect of Nano Zirconium Dioxide (ZrO_2_)-Optimized Content in Polyamide 12 (PA12) and Polylactic Acid (PLA) Matrices on Their Thermomechanical Response in 3D Printing

**DOI:** 10.3390/nano13131906

**Published:** 2023-06-21

**Authors:** Markos Petousis, Amalia Moutsopoulou, Apostolos Korlos, Vassilis Papadakis, Nikolaos Mountakis, Dimitris Tsikritzis, Ioannis Ntintakis, Nectarios Vidakis

**Affiliations:** 1 Mechanical Engineering Department, Hellenic Mediterranean University, Estavromenos, 714 10 Heraklion, Greece; markospetousis@hmu.gr (M.P.); amalia@hmu.gr (A.M.); mountakis@hmu.gr (N.M.); ntintakis@hmu.gr (I.N.); 2 Department of Industrial Engineering and Management, International Hellenic University, Alexander Campus, Sindos, 574 00 Thessaloniki, Greece; apkorlos@ihu.gr; 3 Department of Industrial Design and Production Engineering, University of West Attica, 122 44 Athens, Greece; v.papadakis@uniwa.gr; 4 Department of Electrical & Computer Engineering, Hellenic Mediterranean University, 714 10 Heraklion, Greece; dtsikritzis@hmu.gr

**Keywords:** fused filament fabrication (FFF), 3D printing, nanocomposites, polyamide 12 (PA12), polylactic acid (PLA), zirconium dioxide (ZrO_2_), mechanical properties, material extrusion (MEX)

## Abstract

The influence of nanoparticles (NPs) in zirconium oxide (ZrO_2_) as a strengthening factor of Polylactic Acid (PLA) and Polyamide 12 (PA12) thermoplastics in material extrusion (MEX) additive manufacturing (AM) is reported herein for the first time. Using a melt-mixing compounding method, zirconium dioxide nanoparticles were added at four distinct filler loadings. Additionally, 3D-printed samples were carefully examined for their material performance in various standardized tests. The unfilled polymers were the control samples. The nature of the materials was demonstrated by Raman spectroscopy and thermogravimetric studies. Atomic Force Microscopy and Scanning Electron Microscopy were used to comprehensively analyze their morphological characteristics. Zirconium dioxide NPs showed an affirmative reinforcement tool at all filler concentrations, while the optimized material was calculated with loading in the range of 1.0–3.0 wt.% (3.0 wt.% for PA12, 47.7% increase in strength; 1.0 wt.% for PLA, 20.1% increase in strength). PA12 and PLA polymers with zirconium dioxide in the form of nanocomposite filaments for 3D printing applications could be used in implementations using thermoplastic materials in engineering structures with improved mechanical behavior.

## 1. Introduction

In the past three decades, three-dimensional (3D) printing (3DP) has made significant advancements since its inception, and it is now acknowledged as a highly promising and transformative technology for the manufacturing industry [1,2,3]. Essentially, additive manufacturing (AM) is the broader category of techniques to which 3D printing belongs, where 3D parts composed of diverse materials such as polymers (including thermoplastics [4]), ceramics [5,6], metals [3,7], elastomers [8], thermosets [9], and compounds [10]) are manufactured layer by layer from computer-aided design (CAD) models. Three-dimensional (3D) printing (3DP) has a distinct advantage over traditional manufacturing procedures, such as plastic forming, CNC machining, and injection molding, in producing objects with various materials [11,12], complicated structures [13,14], and customized bulk properties, including thermal [15,16], mechanical [17], antimicrobial [18,19,20], magnetic [21], and catalytic [22] properties, among others. Moreover, 3D printing enables the effortless production of such objects due to their unique nature. As a result, various high-end applications in the biomedical industry have been delivered [23], such as support structures for tissue mechanics [24,25], diagnostic apparatuses in the medical field [26], the fabrication of organs and tissues [27], and in robotics applications, such as 4D-printed structures [28,29], all of which utilize 3DP as the primary production and construction technology in place of conventional manufacturing techniques.

In 1982, the first 3D-printed object was reported by Hideo Kodama [30]. Since then, several 3DP technologies have been formulated for manufacturing objects with different forms and properties, from polymers and metals to ceramics and composites, which depend on the feedstock materials and the process parameters [31]. Some examples of 3DP AM technologies include binder jetting (material jetting), selective laser sintering (powder bed fusion), stereolithography, digital light processing (vat photopolymerization), and Fused Filament Fabrication (FFF) (material extrusion—MEX) [32]. Among them, FFF is commonly used for manufacturing thermoplastic polymeric material components and polymer compounds, such as nano compounds and fiber-reinforced composites [32,33,34,35,36,37,38]. Although significant progress has been made in 3D printing accuracy, automation, and printer parts, such as nozzles and heatable beds, which can affect final part quality [39], research on thermoplastic materials with enhanced mechanical, thermal, electrical, and magnetic properties in comparison to pure polymers is ongoing to create multi-functional printed objects [40].

Polylactic Acid (PLA) is a type of thermoplastic polyester known for its biodegradability and biocompatibility [41]. With its notable mechanical strength and convenient melt-processing characteristics, PLA is extensively employed as a feedstock material in Fused Filament Fabrication (FFF) 3D printing technology [42,43,44]. With a melting point in the range of ~150–160 °C, PLA is not only suitable for various biomedical applications [41,45] but also shows potential for engineering applications [41,46,47]. Therefore, its performance in MEX 3D printing has been extensively reported [41,44,48,49]. It has been documented that PLA serves as a matrix material for MEX 3D printing nanocomposites [47]. Meanwhile, Polyamide 12 (PA12), another engineering thermoplastic, is also highly promising for 3DP applications, particularly in the FFF process [50]. It comes in medical grades, which have also been exploited in MEX 3D printing applications [51]. PA12, being a part of the polyamide family, possesses exceptional attributes including toughness, strength, impact resistance, and resistance to deformation [52,53]. Although extensively used in SLS 3DP processes, PA12 has only recently gained attention in FFF, with studies highlighting its potential for use in rapid prototyping and advanced applications [54], such as structural parts with unique toughness properties. Therefore, the present research is dedicated to investigating the potential of these polymers in FFF 3D printing.

Recently, there has been an extended interest in employing polymeric materials in FFF 3DP, including polymer nanocomposites, as they offer a simple and effective way to improve the properties of 3DP parts [55,56]. Nanoparticles with different geometries, such as spherical (e.g., ZrO_2_, SiO_2_, ZnO, and NPs), tubes (e.g., carbon nanotubes) or wires, and platelet-like (e.g., clays or graphene), at diverse concentrations, were integrated employing the technique of melt-mixing in filaments made of polymeric materials utilized for FFF 3DP [57]. For instance, semicrystalline polymers could be induced to crystallize by nano-inclusions acting as triggering factors, which can have favorable consequences on mechanical characteristics, thermal stability, and more [57]. The incorporation of nanoparticles in polymer matrices has the potential to enhance and expand the properties of 3DP parts for an extensive variety of industrial uses; this includes achieving multifunctional properties such as sensing, actuation, optical properties, and electrical capabilities [58,59,60,61]. Graphene or carbon nanotubes, for instance, have been employed to increase performance in mechanical tests and provide electrical conductivity to 3DP parts [62,63,64,65], indicating the potential of nanoparticle inclusions in polymer matrices. Zirconium dioxide (ZrO_2_) is a biocompatible material and, due to its sufficient chemical and mechanical properties, it is often used in dental applications [66], implants and coatings [67], and optical applications [68]. Due to its popularity, it is applied in 3D printing, almost exclusively in medical treatments for dental and implant applications [69,70], employing the vat photopolymerization and material jetting techniques [71]. Its response in the vat photopolymerization technique has been investigated, mainly toward its effect in the curing process [72,73]. For the MEX 3D printing technique, the literature is still limited [74,75]. Additives used for similar purposes to zirconium dioxide, such as silicon dioxide (SiO_2_), have been employed in vat photopolymerization for dental applications as reinforcement [76,77,78], while zinc oxide (ZnO) has been added to hydrogels and inks for wound healing [79] and electronics [80], respectively.

This research is the first to use ZrO_2_ NPs to reinforce two different types of polar semicrystalline thermoplastics, PA12 and PLA, which have dissimilar macromolecular architectures and side functional groups. The goal is to produce nanocomposite filaments that can be used to create 3D-printed nanocomposite specimens with the material extrusion (MEX) method. As presented in the literature review above, zirconia materials have been employed in additive manufacturing in dental applications, exploiting powder bed extrusion technologies [81]. Scaffolds have been developed [82], and the optical properties of the developed composites have been investigated [83]. On the other hand, the current work investigates the effect of zirconia materials (ZrO_2_) in MEX 3D printing as a reinforcement agent through the development of novel nanocomposites with PA12 and PLA as matrices.

This research analyzes the reinforcement mechanism of two polymeric matrices in 3D-printed samples fabricated with these filaments, and it is found that PA12 exhibits a slightly more substantial enhancement in mechanical characteristics. To examine the fundamental relationship between process, structure, and property for the herein prepared PA12 and PLA ZrO_2_ nanocomposites, the filler loading is maintained at a constant level of 1.0, 2.0, 3.0, and 4.0 wt.% for both cases. All tests conducted for the mechanical, structural, and thermal performance follow the corresponding standards. The morphological characteristics are assessed with Atomic Force Microscopy (AFM) and Scanning Electron Microscopy (SEM). The results show a strong potential for the use of the popular ZrO_2_ as a reinforcement agent in MEX 3D printing, providing nanocomposites with robust mechanical strength compared to the pure matrices. Such a performance further expands the application fields of the MEX 3D printing process. Still, the reinforcement effect differs between the two polymers tested, showing that the findings presented herein cannot be generalized for other polymers and AM processes.

## 2. Materials and Methods

### 2.1. Raw Materials Employed for the 3D Printing Process

PA12, provided by Arkema S.A. of Colombes, France, in fine pellet form, is a rigid polyamide with a high viscosity of AESNO TL grade. Its specifications from the supplier include a melting temperature of 180 °C (as per ISO 11357-3), a melt volume flow rate (MVR) of 8.0 cm^3^/10 min at 235 °C/5.0 kg, a density of 1.01 g/cm^2^ (as per ISO 1183), and Vicat softening temperature of 142 °C (as per ISO 306/B50). PLA, on the other hand, is a biodegradable and biocompatible polymer that has been gaining popularity in various industrial applications. Plastika Kritis SA of Heraklion, Crete, Greece, provided the primary material, in powder form as a thermoplastic material, under the trade name Ingeo Biopolymer 3052D grade, featuring a 116,000 g/mol molecular weight, as per the supplier’s specifications. Its use in mechanical, biological, and biomechanical applications is increasingly being explored. The manufacturing process of the materials created in this study used polymer as the matrix material, and the addition of nanocomposite material was manufactured using the appropriate melt-mixing process, simultaneously without using heat stabilization, lubrication, and UV stabilizer fillers, to assess the effect of the filler only on the matrices. Nano zirconium dioxide (ZrO_2_) was the additive herein. It is a very stable nano metal oxide (ceramic). The employment of this material in a variety of applications is the research area of this study because it is a material that also has high acid and alkali resistance, corrosion resistance, and high-temperature resistance. It was sourced from Nanographi (Ankara, Turkey), having 99.95% purity and a 30 nm particle size.

### 2.2. Filament Preparation and 3D Printing Parameters

**Figure 1:**
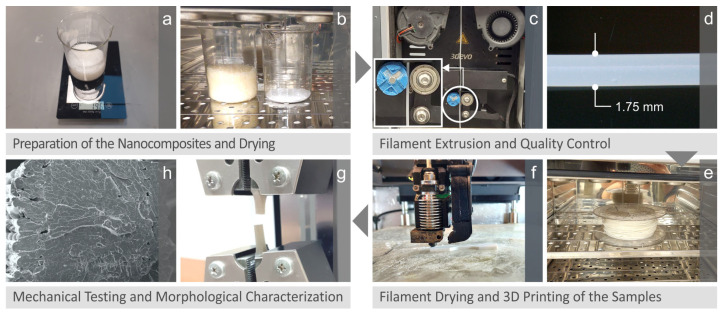
Preparation of the nanocomposites and mechanical testing: (**a**) raw material’s weight, (**b**) drying process for the raw materials, (**c**) filament creation using extrusion, (**d**) quality control of the filament, (**e**) drying process of the filament, (**f**) creating samples using 3D printing, (**g**) tensile test for mechanical characterization, (**h**) image of the fracture surface acquired with SEM.

Initially, raw materials were turned into 3D printing filaments. An incremental methodology was used to accomplish the most feasible process for the distribution of the zirconium dioxide additive in the parent material. The approach followed in this paper is depicted in Figure 1. In detail, firstly, from the raw materials procured from the above producers, weighing was carried out using a precision balance (Figure 1a). Then, it was deemed necessary to dry the raw materials (Figure 1b), and the next step was to create the filament through the extrusion process with the corresponding quantities from the materials to obtain the corresponding research quotas (Figure 1c). After the filament was created, inspection was necessary, and quality control was carried out. The diameter dimension of the filament after extrusion was measured (Figure 1d). The generated filament was gathered into a bobbin and then taken to a dryer to dry it in preparation for 3D printing (Figure 1e). The filament diameter dimension (1.75 mm) was chosen to be suitable for the available 3D printer, on which the corresponding specimens with different compounds were manufactured. The materials were mixed in a single screw extruder (Composer 450, 3D Evo B.V., Utrecht, The Netherlands). After printing the specimens (Figure 1f), the material properties had to be determined through appropriate mechanical tests in order to characterize the whole 3D printing result. Figure 1g shows a snapshot of a tensile test, and other tests were carried out and discussed later on. The final step in the research was a Scanning Electron Microscopy (SEM) of the specimen’s morphological characterization before and after fracture from the tests performed.

**Figure 2:**
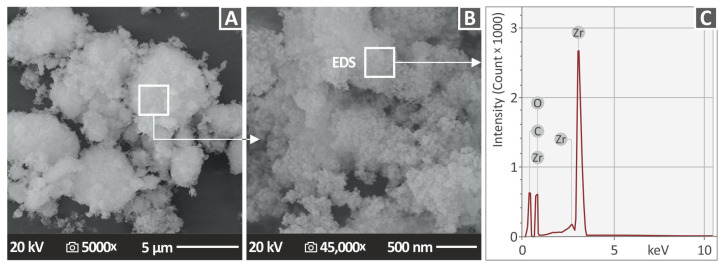
SEM photos of ZrO_2_ powder at (**A**) magnification 5000×, (**B**) 45,000× magnification, and (**C**) EDS compositional analysis from the ZrO_2_ powder.

In Figure 2, zirconium dioxide (ZrO_2_) powder’s SEM photos at two diverse magnifications are presented (Figure 2A: 5000× and Figure 2B: 45,000×), together with energy-dispersive analysis (EDS) (Figure 2C). The nanocrystallites’ morphology can be clearly observed using SEM analysis to capture the shape of the zirconium dioxide (ZrO_2_) nanoparticles. In Figure 2C, the Zr element in the nanomaterial is shown as a peak that dominates the EDS spectrum analysis. Using the SEM images provided the size of the nanoparticles. In the EDS plots, carbon is an element present in organic materials since the parts printed are made of polymeric materials. Zirconium dioxide (ZrO_2_) powder was used with a sputtering process using carbon to be observed by the SEM equipment (Figure 2C). This is why carbon is presented in the EDS plot, although carbon is not a material of zirconium dioxide (ZrO_2_) powder elements. It is worth noting that EDS analysis highlights the elements in the part being tested, while any material additions for material preparation will be visible in the final results, regardless of whether they do not belong to the original part produced by 3D printing. This process is not accurate for the calculation of element concentration because the measurement procedure focuses on a specific point of the specimen. The analysis of the elements in the part’s area is captured in the correct way, and the peaks are highlighted, while the rest of the results are more qualitative than quantitative. Therefore, the results for the elements, Zr and O, could not be accurate using the EDS method. More advanced methods should have been used to obtain more accurate results.

The 3D printing procedure was conducted utilizing an Intamsys Technology Co. Ltd-provided Funmat HT 3D printer (located in the city of Shanghai in China) for the fabrication of the specimens from PA12, PLA, PA12/ZrO_2_, and PLA/ZrO_2_ materials. At first, the determination of the optimum 3D printing parameters was necessary to be carried out in order for the 3D printing result to be the best. The selection of the optimum parameters was carried out by 3D printing a sufficient number of specimens with different 3D printing parameters for the cases of PA12, PLA, and nanoparticles. In previous research, the authors of this paper investigated the fabrication of the filament using the extrusion method and also the optimum 3D printing factors for PA12 and PLA [47]. Finally, the following parameters were used for PA12 and PA12/ZrO_2_ nanocomposites: layer height, 0.20 mm; 45-degree raster angle; nozzle temperature, 270 °C; bed temperature, 90 °C; filling density, 100%; printing speed, 40 mm/s; construction of 4 layers in the region of the specimen adjacent to the 3D printing bed; and construction of 2 layers in the upper part of the specimen, while for PLA and PLA/ZrO_2_ nanocomposites: 45-degree build angle; layer height, 0.20 mm; nozzle temperature, 210 °C; bed temperature, 50 °C; filling density, 100%; 3D printing speed, 40 mm/s; construction of 4 layers in the area of the specimen adjacent to the 3D printing bed; and construction of 2 layers in the upper part of the specimen.

### 2.3. Experimental Details

Material characterization was performed for before and after fracture specimens using Scanning Electron Microscopy (SEM). The equipment used was a field-emission SEM model (JSM-IT700HR Jeol Ltd., Tokyo, Japan). The analysis was carried out under specific conditions, including a 20 kV acceleration voltage and high-vacuum mode. The characterization of the specimens was performed using a secondary electron (SE) detector. Before the introduction of the 3D-printed specimens into the SEM, sputter coating Au was used for the preparation of the specimens (5 nm thin film).

Raman spectroscopy was achieved using a LabRAM HR Raman spectrometer, which was manufactured by HORIBA Scientific in Kyoto, Japan. The system integrates a solid-state laser module operating at a wavelength of 532 nm, capable of delivering a maximum output power of 90 mW. Delivery of light and collection of the Raman signal was performed with an Olympus objective lens (LMPlanFL N, Olympus, Tokyo, Japan). The system utilized a microscope with a numerical aperture of 0.5, providing 50× magnification and a working distance of 10.6 mm. To ensure safe laser power, a neutral density filter with 5% transmittance was employed, limiting the sample to 2 mW. The microscope offered a lateral resolution of 1.7 μm and an axial resolution of 2 μm. The Raman spectral resolution was approximately 2 cm^−1^, achieved through a grating with 600 grooves. The measurement Raman spectral range extended from 50 to 3900 cm^−1^, necessitating the use of three consecutive optical windows. For every measurement point, an exposure time of 5 s with 5 accumulations was used.

**Figure 3:**
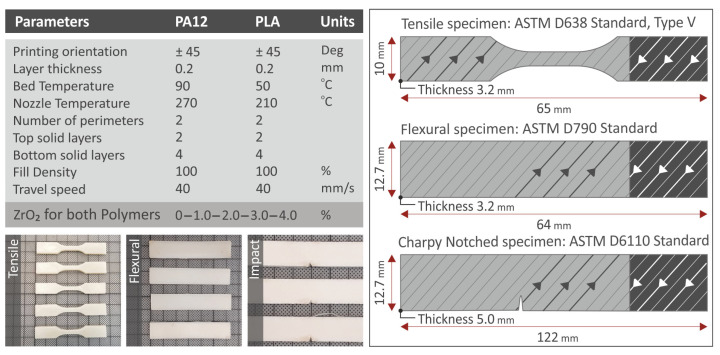
The 3D printing factors used for specimen manufacturing and the geometry of the samples for tension, 3-point bending, and impact tests.

For mechanical property determination, it was considered necessary to perform tensile, three-point bending, and notch impact toughness tests on the specimens. Figure 3 illustrates the dimensions of the specimens utilized in each test. The temperature of the laboratory in which the tests were carried out was measured and kept constant at 23 °C. The specimens for tensile testing had dimensions of 65 mm length, 10 mm width, and 3.2 mm thickness, and the ASTM D638 Standard, Type V, was followed. Imada MX2 machine (Imada Inc., Northbrook, IL, USA) was used for tensile testing, and the elongation rate during the test was set constant at 10 mm/min.

The three-point bending experiments were executed in accordance with the international standard ASTM D790. The geometry of the specimens had dimensions as illustrated in Figure 3, i.e., specimen length, 64.0 mm; specimen width, 12.7 mm; and specimen thickness, 3.2 mm. For proper support of the specimen and the support rollers in the 3-point bending machine, rollers were set at a 52.0 mm distance. Flexural tests were conducted using an Imada MX2 machine (provided by Imada Inc. in Northbrook, IL, USA) with a set elongation rate of 10 mm/min. The Charpy investigations were performed in accordance with the international ASTM D6110 Standard. The notched samples had a 5 mm thickness, 12.7 mm width, and 122 mm length (Figure 3). A Charpy impact machine, Terco MT 220 (Terco, Huddinge, Sweden), was used in the impact tests. In this paper, for all the tests conducted, six (6) specimens were evaluated for the pure, as well as for the 3DP PA12/ZrO_2_ and PLA/ZrO_2_, nanocompounds in order to evaluate the results statistically. For the test results, the mean values were reported with the standard deviation results.

Finally, an ASTM E384-17 was used for the microhardness measurements, which were carried out for both PA, PLA, and nanoparticle materials. The sample’s surface was polished before using for the measured procedure thoroughly. A testing machine (model name, 300-Vickers, provided by the Innovatest Europe BV company, located in Maastricht, The Netherlands) was used. The test settings were 100 gF for the force and 10 s duration of indentation during the test. Imprints were carried out for six (6) different samples of PA12, PLA, and nanocomposite materials, and the corresponding measurements were performed.

## 3. Results

### 3.1. TGA Examination of Neat PA12, PLA, PA12/ZrO_2_, and PLA/ZrO_2_ Nanocomposites

**Figure 4:**
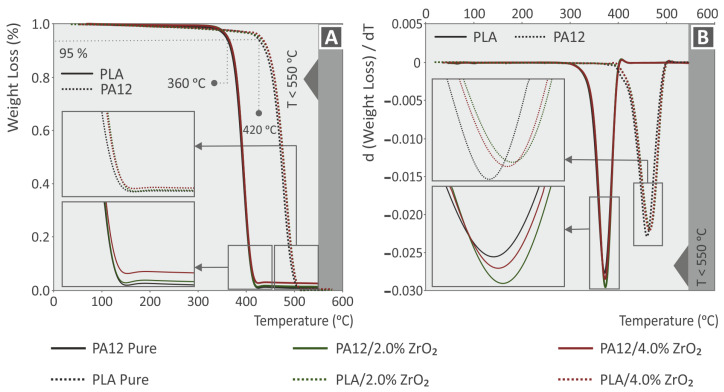
Compounds (**A**) thermogravimetric analysis and (**B**) derivative thermogravimetry graphs, acquired from pure Polyamide 12 and Polylactic Acid, and their ZrO_2_ nanocompounds at 2.0 and 4.0 wt.% filler loadings.

TGA analysis is considered important in the case of plastics and nanoparticle additives in order to investigate the stability of the polymer under thermal loading. To conduct this investigation, a series of experiments were performed under a nitrogen atmosphere. The results of these experiments are presented in Figure 4, which highlights the nature of the diverse nanocompounds with the precise filler quantities in each sample, while both the polymer matrices were completely decomposed. Figure 4A displays the thermogravimetric analysis (TGA), while Figure 4B shows the results for the weight loss rate. PA12 has greater thermal stability in comparison to PLA. PA12′s onset temperature of decomposition ($T_{on}^{d}$) was found to be 420 °C, and the respective value for the PLA polymer was measured at 330 °C (Figure 4A). These two polymeric materials (PA12 and PLA) completely decomposed at temperatures above 500 °C, and the remaining material in certain curves relates to the ZrO_2_ nanoparticle material loading. The results of the ZrO_2_ nanoparticle quantification results confirmed the remaining mass through the TGA [84].

The addition of ZrO_2_ nanofillers had a minor impact on the stability of both polymers under thermal loading, as evidenced by the TGA and DTG curves. The $T_{on}^{d}$ value slightly increased, indicating that the nano compounds’ stability in the thermal tests was marginally increased by the presence of ZrO_2_ nanoparticles. The response observed in the DTG diagrams was slightly different. In PLA, the higher weight loss ratio shifted to marginally increased temperatures, and the rate soared with the addition of ZrO_2_ nanoparticles. With regard to PA12, the higher weight loss ratio occurred at marginally higher temperatures. The rate decreased vaguely with the addition of ZrO_2_ nanoparticles. However, the differences were not significant and were likely due to the interactions between the filler and the polymers. This study’s selected temperatures for polymer processing were much lower than the pure polymer matrices’ decomposition temperatures, and this was confirmed by the TGA and DTG analyses.

### 3.2. Investigation through Raman and EDS of Unfilled PA12, PLA, PA12/ZrO_2_, and PLA/ZrO_2_ Nanocomposites

**Figure 5:**
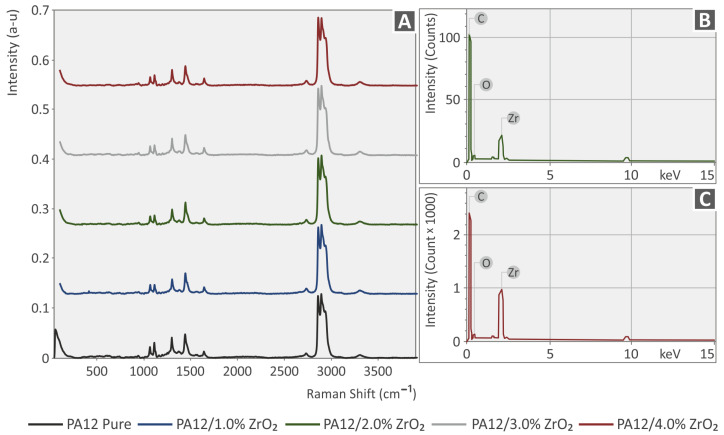
(**A**) Spectra acquired with Raman for the unfilled Polyamide 12 with 1.0 wt.% ZrO_2_, Polyamide 12 with 2.0 wt.% ZrO_2_, Polyamide 12 with 3.0 wt.% ZrO_2_, and Polyamide 12 with 4.0 wt.% ZrO_2_. (**B**,**C**) EDS curves acquired from the 3DP Polyamide 12/ZrO_2_ nanocomposites.

**Figure 6:**
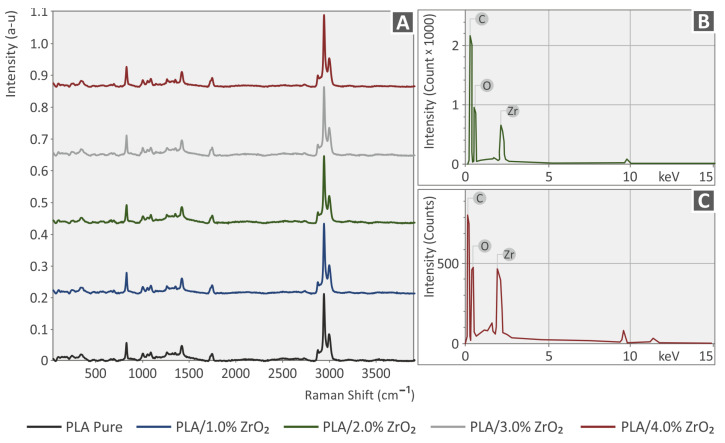
(**A**) Spectra acquired with Raman for the unfilled Polylactic Acid, Polylactic Acid with 1.0 wt.% ZrO_2_, Polylactic Acid with 2.0 wt.% ZrO_2_, Polylactic Acid with 3.0 wt.% ZrO_2_, and Polylactic Acid with 4.0 wt.% ZrO_2_. (**B**,**C**) EDS curves acquired from the 3DP Polylactic Acid/ZrO_2_ nanocomposites.

In Figure 5 and Figure 6, the clear Raman spectra are depicted from the pure PLA and PA12 materials and the PLA/ZrO_2_ PA12/ZrO_2_ compounds. There are no significant Raman spectral differences from the ZrO_2_ additive. All differences observed were close to the noise level (<1%). The related Raman peaks from the PLA and PA12 pure samples are presented in Table 1 and Table 2, together with their assignments validated by the literature.

### 3.3. Metrology for the MEX 3D Printing Filament Diameter Employing Optical Means

It is known that 3D printer manufacturers have standardized the diameters of the filament they use for FFF 3D printing. In the case under consideration, a 3D printer was used operating with a 1.75 mm filament. For this reason, it was necessary to use an extruder with a 1.75 mm die. In this paper, the 3D Evo provided the single screw employed, which had the model name Composer 450 (manufactured by 3D Evo B.V. in Utrecht, The Netherlands). Due to the increased temperature and, subsequently, the cooling of the filament, it shows contraction and fluctuation in the diameter dimension, which makes the filament then difficult to be processed in 3D printing. It is important for 3D printing to produce a filament with as constant a diameter as possible. It is important to mention that in order to achieve a high-level result during 3D printing, a filament of high quality, i.e., roundness, constant diameter, and regular distribution of particles, is an essential requirement for stable and high-quality 3DP parts. It is worth commenting that the stability of the filament diameter directly affects the 3D printing result since it is input as data to the slicer software, which programs the 3D printing, and therefore, any non-uniformity will bring about negative effects on the dimensional accuracy of the 3D-printed object.

**Figure 7:**
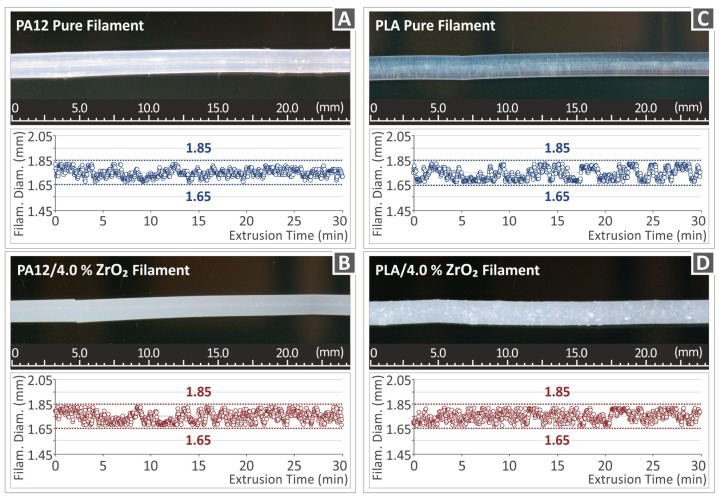
Metrology for the filament diameter of (**A**) optical microscope images for PA12 pure filament, (**B**) optical microscope images for PA12/ZrO_2_ (4.0 wt.%), (**C**) stereoscope images for PLA pure filament, (**D**) stereoscope images for PLA/ZrO_2_ (4.0 wt.%).

The extruder’s peculiarity and a great advantage is advanced technology since it contains an integrated sensor, which records, in real-time, the diameter of the filament during the extrusion process. Figure 7 illustrates the monitored real-time diameter of the filament of pure polymeric matrices PA12 (Figure 7A) and PLA (Figure 7C), as well as the two highly charged nanocompounds, i.e., Polyamide 12/ZrO_2_ with 4.0% weight-to-weight concentration (Figure 7B) and Polylactic Acid/ZrO_2_ with 4.0% weight-to-weight concentration (Figure 7D) during a total extrusion time of 30 min to generate enough filament for the experiments. The sensor recorded a filament diameter in real-time, which fell within the acceptable range of 1.75 mm ± 0.10 mm. This was achieved by adjusting the extrusion speed during the process to ensure a consistent diameter along the entire length of the filament, thereby meeting the necessary accuracy tolerances. Microscope and stereoscope images of the lateral surface of the filament for all the nanocomposites created in this study showed a smooth surface without any defects or voids, indicating that the filament quality was good. These results suggest that the factors used in this study were suitable [91].

### 3.4. AFM Evaluation of Surface Roughness for 3D Printing Filaments Containing Neat Polymers and Nanocomposites

**Figure 8:**
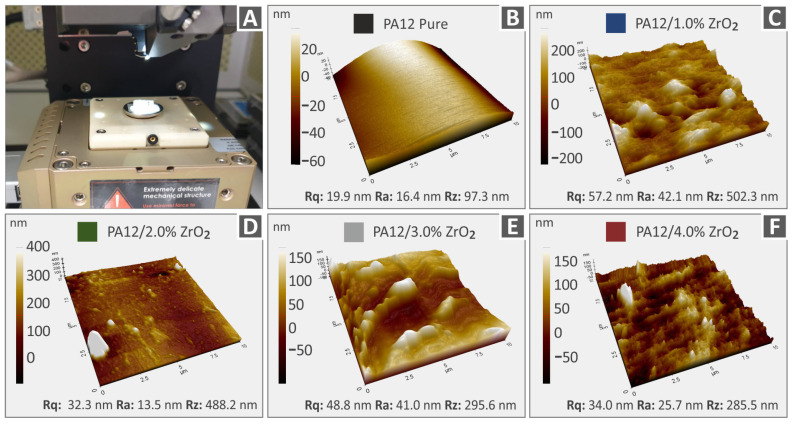
AFM surface topography photos and the Rq, Ra, and Rz respective roughness values of PA12 and PA12/ZrO_2_ nanocompound filaments. (**A**) AFM setup, (**B**) unfilled Polyamide 12, (**C**) Polyamide 12/ZrO_2_ 1.0 wt.%, (**D**) Polyamide 12/ZrO_2_ 2.0 wt.%, (**E**) Polyamide 12/ZrO_2_ 3.0 wt.%, (**F**) Polyamide 12/ZrO_2_ 4.0 wt.%.

**Figure 9:**
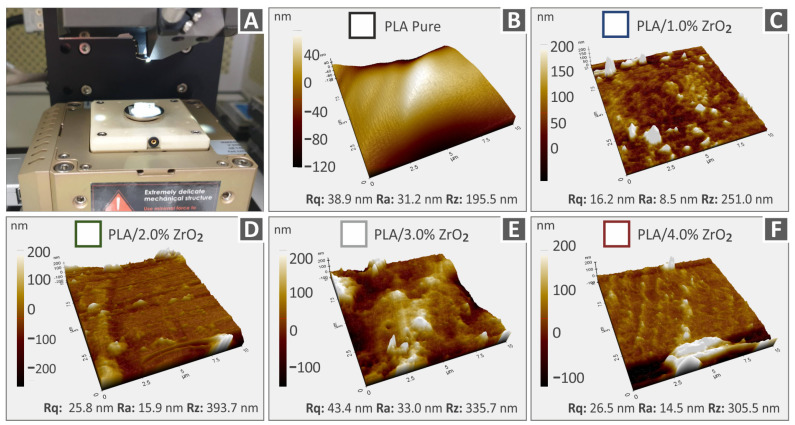
AFM topography photos and the respective Rz, Rq, and Ra roughness values of Polylactic Acid and Polylactic Acid/ZrO_2_ nanocompound filaments. (**A**) AFM setup, (**B**) pure Polylactic Acid, (**C**) Polylactic Acid/ZrO_2_ 1.0 wt.%, (**D**) Polylactic Acid/ZrO_2_ 2.0 wt.%, (**E**) Polylactic Acid/ZrO_2_ 3.0 wt.%, (**F**) Polylactic Acid/ZrO_2_ 4.0 wt.%.

Measuring the roughness of a mechanical part is crucial in 3D printing because it quantifies the surface marks produced during manufacturing. Among the commonly utilized roughness parameters are Rq, representing the root-mean-square roughness; Ra, denoting the mean roughness; and Rz, indicating the disparity between the highest peak and lowest valley on the surface. Figure 8 and Figure 9 depict the AFM 3D topography images that were obtained from various extruded 3DP filaments in the study. Figure 8A and Figure 9A showcase the AFM setup used to measure specimens fabricated with different material percentages (0–1–2–3–4 wt.%) and also with different materials—PA12 and PLA. Figure 8 displays the topography pictures along with the associated derived surface roughness values for Rz, Rq, and Ra of different filament compositions, namely, Polyamide 12 (Figure 8B), Polyamide 12/ZrO_2_ with 1.0% weight-to-weight concentration (Figure 8C), PA12/ZrO_2_ 2.0 wt.% (Figure 8D), Polyamide 12/ZrO_2_ with 3.0% weight-to-weight concentration (Figure 8E), and Polyamide 12/ZrO_2_ with 4.0% weight-to-weight concentration (Figure 8F). Similarly, Figure 9 presents the topography images and corresponding roughness values of Polylactic Acid (Figure 9B), Polylactic Acid/ZrO_2_ with 1.0% weight-to-weight concentration (Figure 9C), Polylactic Acid/ZrO_2_ with 2.0% weight-to-weight concentration (Figure 9D), Polylactic Acid/ZrO_2_ with 3.0% weight-to-weight concentration (Figure 9E), and Polylactic Acid/ZrO_2_ with 1.0% weight-to-weight concentration (Figure 9F). This study’s findings reveal that, in each case, the surface roughness rises as ZrO_2_ nanoparticles are added to and increased in concentration in the corresponding polymer matrix. It is proposed that this rise in surface roughness may be due to the presence of nanoparticles on the filament’s surface and the flow behavior of the nanostructured material differs when subjected to different conformations of PA12 and PLA polymer chains, in contrast to the behavior observed in the pure polymeric materials [83,92,93,94,95].

The incorporation of ZrO_2_ additive results in different impacts on the surface roughness of the nanocomposites. In comparison to pure PLA, pure PA12 exhibits a smoother surface. However, when PA12 is utilized as the matrix material for the nanocomposites, the surface roughness increases in comparison to their PLA counterparts. Nevertheless, the observed differences in surface roughness are minimal and not considered significant. The inclusion of the zirconium dioxide filler has a distinct impact on each thermoplastic’s structure. Additionally, as the measurements were made at random locations, variations are expected, partly as a result of the area’s microscale topography.

### 3.5. Mechanical Characterization of the Produced Filaments for MEX 3DP and the Respective 3DP Samples: Unfilled Polyamide 12, Polylactic Acid, and Their ZrO_2_ Nanocompounds

**Figure 10:**
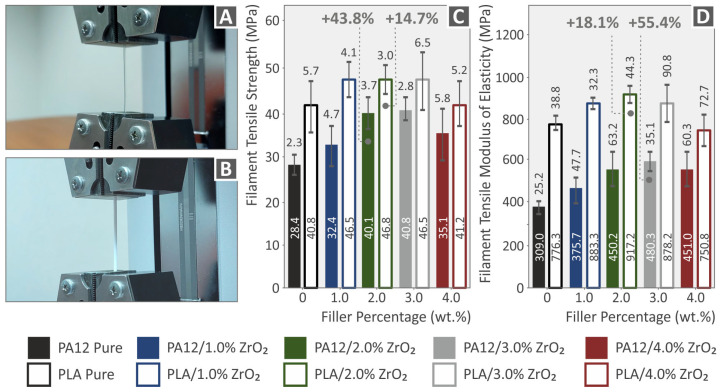
(**A**,**B**) Typical polymer filaments for the neat and nanocomposite materials being tested; (**C**,**D**) the tensile strength and modulus of elasticity of 3D-printed filaments made from PA12, PLA, and nanocomposites containing different weight percentages (1.0, 2.0, 3.0, and 4.0 wt.%) of ZrO_2_ filler. The legend provides information on the colors assigned to each material in the graphs.

In this study, the tensile properties of neat PA12, PLA, and nanocompound filaments were tested at two stages: first at the filament level and then on 3DP dog-bone tensile test specimens. Figure 10A,B depict a correspondent neat polymer filament and a nanocompound filament, respectively. This study’s findings for the tensile tests (strength and modulus of elasticity) regarding the extruded and produced filaments are presented in Figure 10C, and D, respectively, which show mean values and corresponding standard deviations. This research reveals that the addition of ZrO_2_ NPs had a beneficial reinforcing influence for different additive loadings in all extruded filaments. Specifically, the ultimate increase in the samples’ strength was observed at 2.0 wt.% for both Polyamide 12 (43.8%) and Polylactic Acid (14.7%). Regarding the modulus of elasticity, the highest increase was detected at 3.0 wt.% for PA12 (18.1%) and 2.0 wt.% for PLA (55.4%) [96,97,98,99,100].

**Figure 11:**
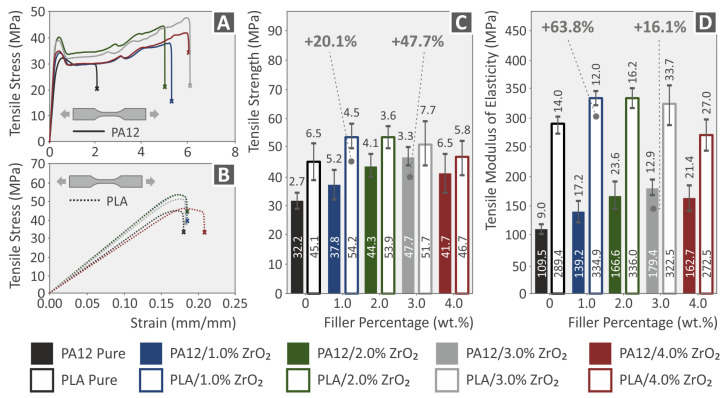
Tensile experiment findings, stress (MPa) vs. strain (%) graphs for (**A**) PA12 and PA12/ZrO_2_ nanocompounds, (**B**) Polylactic Acid and Polylactic Acid/ZrO_2_ nanocompounds; (**C**) tensile strength; (**D**) tensile modulus of elasticity for 3D-printed Polyamide 12, Polylactic Acid, and all nanocompounds at 1.0 wt.%, 2.0 wt.%, 3.0 wt.%, and 4.0 wt.% ZrO_2_ filler loading. Each graph’s legend identifies the color that corresponds to each material.

The PA/ZrO_2_ polymers showed an increase in tensile strength for 1, 2, and 3 wt.% concentration of the ZrO_2_ filler, followed by a decrease with the further addition of ZrO_2_ in PA. The PLA/ZrO_2_ polymers showed an increase in tensile strength up to 1 wt.% concentration of the ZrO_2_ filler, followed by a decrease with a further increase in concentration. However, even at 4 wt.%, the strength compared to both polymeric materials in pure form in the tensile test was increased. The effect of the enhancement was substantially stronger in PA12 compared to PLA. Figure 11A,B show comparative tensile stress (MPa) vs. strain (%) graphs for both polymeric materials and their nanocompounds (PA12, PA12/ZrO_2_, PLA, and PLA/ZrO_2_). In Figure 11C,D, the tensile test findings regarding the strength and modulus of elasticity values are reviewed for all 3DP tensile specimens. ZrO_2_ NPs showed a positive reinforcement effect for all samples fabricated with the 3D printing process, with the greatest increase observed at 1.0 wt.% for PLA (20.1% and 63.8%, respectively) and at 3.0 wt.% for PA12 (47.7% and 16.1%, respectively) [100,101,102,103].

The way in which the pure 3DP polymeric materials respond to stress and strain is similar to what we observed in a previous study we conducted. In that study, we carefully examined how the tensile characteristics (stiffness and strength) of various polymeric materials commonly utilized in Fused Filament Fabrication 3D printing were affected by the rate of strain [104].

**Figure 12:**
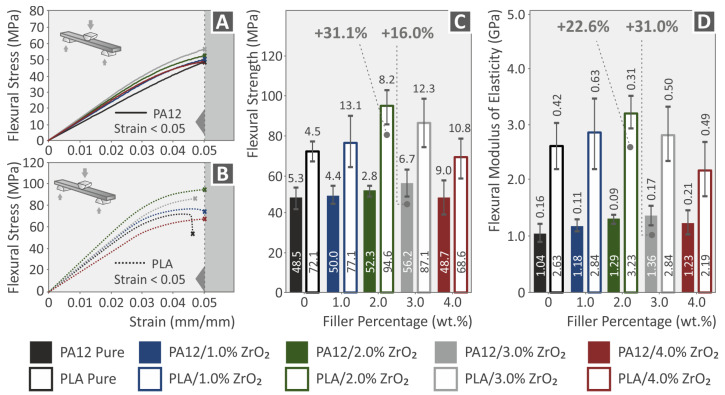
Graphs showing the relationship between flexural stress (MPa) and strain (%) for (**A**) PA12 and its ZrO_2_ nanocompounds, and (**B**) PLA and its ZrO_2_ nanocompounds. (**C**,**D**) The average values and standard deviations in flexural strength and flexural modulus for 3D-printed Polyamide 12, Polylactic Acid, and all nanocompounds/ZrO_2_ additive loading. In each graph, the legend indicates the color which corresponds to each material.

The findings of the flexural tests performed on pure PA12 and PLA materials and their ZrO_2_ nanocomposites were analyzed and are presented in Figure 12. The flexural stress vs. strain curves for all specimens was analyzed according to specific ASTM standards and are shown in Figure 12A,B. A summary of the flexural test outcomes (flexural strength and flexural modulus of elasticity) for all 3DP samples is provided in Figure 12C,D. The inclusion of ZrO_2_ nanoparticles exhibited a beneficial effect on the flexural characteristics of both PA12 and PLA nanocomposites. The greatest improvements in flexural strength and modulus were observed at 3.0 wt.% for PA12 (16% and 31% increase, respectively) and at 2.0 wt.% for PLA (31.1% and 22.6% increase, respectively). The tensile testing results aligned with the increasing trend in flexural modulus and strength parameters for PA12 and PLA composites. Even the nanocomposites with the lowest flexural strength values surpassed the corresponding values of the pure polymers for volumes up to 3 wt.%. Therefore, the addition of ZrO_2_ clearly ameliorated the flexural properties of the polymers [105,106].

**Figure 13:**
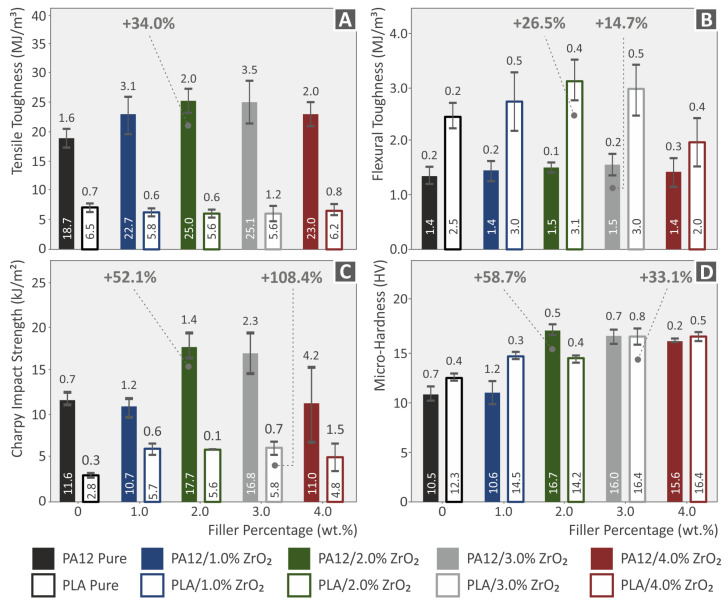
The toughness properties (**A**) under tensile loading, flexural loading (**B**), Charpy’s notched impact strength in kJ/m^2^ (**C**), and Vickers micro-hardness (HV) (**D**) of the pure PA12 and PLA materials, as well as their respective nanocompounds/ZrO_2_ filler loading were analyzed and presented in graphical form. The legend indicates the color that corresponds to each material in the bars.

Figure 13 displays the mechanical properties of neat PA12 and PLA, including tensile toughness (Figure 13A), flexural toughness (Figure 13B), impact strength (Figure 13C), and micro-hardness measured using the Vickers scale (HV) (Figure 13D), as well as their corresponding nanocompounds with ZrO_2_ filler loading at 1.0, 2.0, 3.0, and 4.0 wt.%. This research demonstrates that the material’s resistance to fracture was enhanced by the inclusion of ZrO_2_ nanoparticles because they effectively inhibit the initiation, development, and propagation of cracks when subjected to quasi-static tensile or flexural mechanical stress fields. The highest increase in tensile toughness was noted in PA12/ZrO_2_ (2.0 wt.%) nanocomposites with a 34% increase. Regarding Polylactic Acid/ZrO_2_ nanocomposites, a slight decrease was found. Similarly, the highest increase in flexural toughness was observed in Polyamide 12/ZrO_2_ (3.0 wt.%, 14.7%) and Polylactic Acid/ZrO_2_ (2.0 wt.%) with a 22.5% rise. The best response for impact strength was Polyamide 12/ZrO_2_ (2.0 wt.%) with a 52.1% rise and Polylactic Acid/ZrO_2_ (3.0 wt.%) with a 108.4% increase in the impact strength value, while Vickers micro-hardness exhibited a similar pattern, with Polyamide 12/ZrO_2_ (2.0 wt.%) showing a 58.7% rise and Polylactic Acid/ZrO_2_ (3.0 wt.%) showing a 33.1% rise [104,107,108].

The results demonstrate that the impact, toughness-related, and Vickers micro-hardness findings depicted satisfactory performance. These properties exhibit an increasing trend and a more favorable effect as the filler loading increases. The material’s capacity to withstand greater mechanical energy before fracture is attributed to a mechanism associated with cracking. However, the pure tensile and flexural characteristics, including strength and modulus of elasticity, are influenced by a mechanical percolation threshold attained or generated at additive loadings ranging from 1.0 to 2.0 wt.%.

### 3.6. SEM Morphological Analysis of the Side Surface of the 3D-Printed Samples and the Tensile Test Specimen Fractured Surfaces

**Figure 14:**
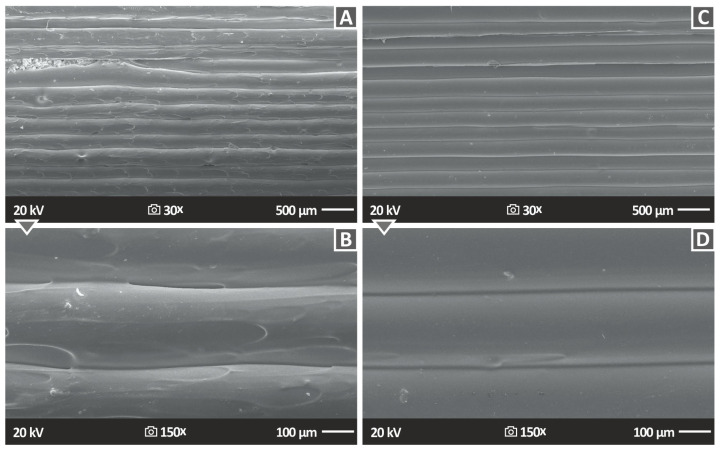
Photos acquired with SEM of the side surface morphological characteristics of (**A**) PA12 2.0% ZrO_2_ 30× side 500 μm, (**B**) PA12 2.0% ZrO_2_, (**C**) PA12 4.0% ZrO_2_ 30× side 500 μm, (**D**) PA12 4.0% ZrO_2_ 150× side 100 μm.

Figure 14 shows the lateral surface morphology of 3DP PA12/ZrO_2_ 2% and PA12/ZrO_2_ 4% nanocompounds, indirectly emphasizing the external structure of the 3DP specimens. This is clearly visible in the figures and is essentially the product of the 3D-printed layers and the underlying fusion between them. Additionally, Figure 14 shows, at two different magnifications, namely, 30× (Figure 14A,C) and 150× (Figure 14B,D), the lateral morphology of the specimens. All samples observed excellent structuring and fusion between layers, which emphasizes the great quality of the produced 3DP raw material filaments. Additionally, due to the structure of this part, it was confirmed that the 3D printing factors chosen for the 3D printing of the samples in this research work were optimal. Moreover, it was observed that there were no discontinuities, gaps, cracks, etc., between the layers; in the event that such a scenario occurs, it would result in the creation of parts where the layers possess weak inter-facial shear strength. Consequently, this would lead to 3D-printed objects with diminished mechanical performance.

**Figure 15:**
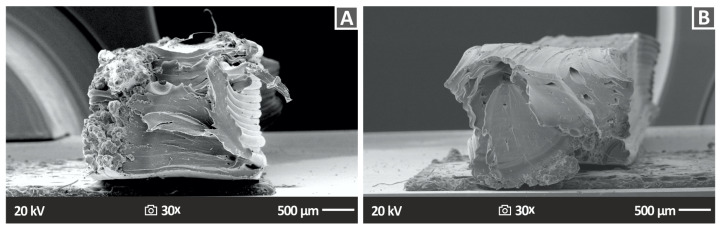
Photos acquired with SEM of the tensile specimens’ fractured surface morphological characteristics (**A**) PA12 2.0% ZrO_2_ 30× fracture 500 μm, (**B**) PA12 4.0% ZrO_2_ 30× fracture 500 μm.

The SEM images corresponding to the fractured surfaces revealed the findings of the fractography analyses of the tensile specimens and are displayed in Figure 15 (PA12/ZrO_2_ nanocomposites 2% and 4 wt.%). Similar results were obtained for the other cases. For the nanocomposites, in all cases, except for PA12/ZrO_2_ 2.0 wt.% (Figure 15A), observations indicate the presence of a relatively ductile fracture mechanism, characterized by rough fracture surfaces and the presence of 3D-printed filaments on the fractured surface (Figure 15B). However, the general conclusion is that it is impossible to identify any individual components among the various additively created layers, neither gaps between layers nor within layers, which is an indication of high-quality printing between layers and the optimal 3DP manufacturing parameters chosen in this research [108,109].

**Figure 16:**
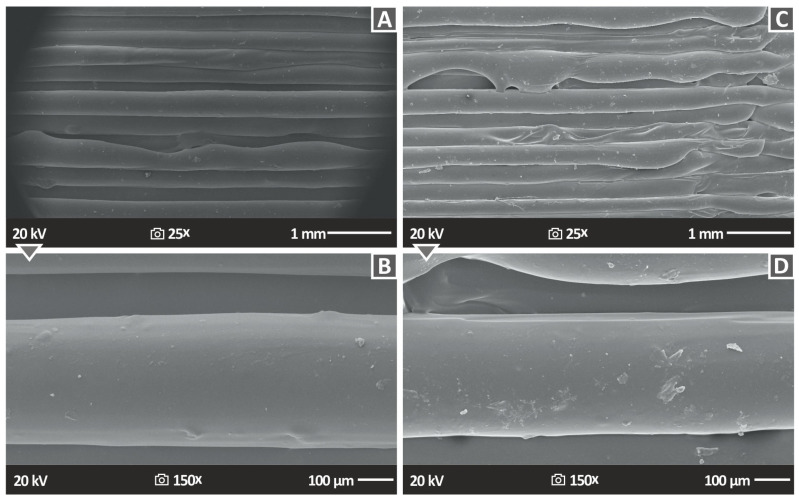
SEM photos of the lateral surface morphology of (**A**) PLA 2.0% ZrO_2_ 25× side 1 mm, (**B)** PLA 2.0% ZrO_2_ 150× side 100 μm, (**C**) PLA 4.0% ZrO_2_ 25× side 1 mm, (**D**) PLA 4.0% ZrO_2_ 150× side 100 μm.

Figure 16 shows the lateral surface morphology of 3DP PLA/ZrO_2_ 2 wt.% and PLA/ZrO_2_ 4 wt.% nanocompounds, indirectly emphasizing the external structure of the 3DP specimens. Additionally, Figure 16 shows, at two different magnifications, namely, 25× (Figure 16A,C) and 150× (Figure 16B,D), the lateral morphology of the specimens. All samples observed excellent structuring and fusion between layers, which emphasizes the great quality of the produced 3DP raw material filaments. Additionally, due to the structure of this part, it was confirmed that the 3D printing factors chosen for the 3D printing of the samples in this research work were optimal, also in the case of PLA. The same conclusions as for PA12 can be drawn in the case of PLA. The layers do not have a uniform shape throughout their length, as shown, attributed to the addition of ZrO_2_ in the matrix. The 3D printing settings were not optimized for the nanocompounds. They were the same as the unfilled matrix to have comparable results.

**Figure 17:**
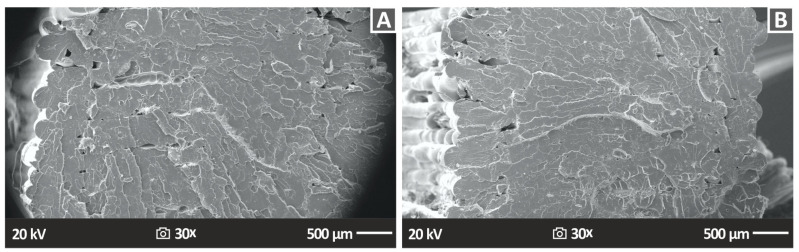
SEM photos of the morphology of the tensile samples’ fractured surface (**A**) PLA 2.0% ZrO_2_ 25× fracture 1 mm, (**B**) PLA 4.0% ZrO_2_ 25× fracture 1 mm.

The SEM images corresponding to the fractured surfaces that revealed the findings of the fractography analyses of the tensile specimens are displayed in Figure 17 (PLA12/ZrO_2_ nanocomposites 2% and 4 wt.%). Similar results were obtained for the other cases. For the nanocomposites ZrO_2_, in every instance, a comparatively ductile fracture mechanism with coarse fracture surfaces and 3DP filaments on the fractured surface could be observed (Figure 17A,B). Nevertheless, the overall consensus is that, in all cases, there are no discernible boundaries between the various layers produced through additive manufacturing, neither gaps nor voids, which is an indication of high-quality 3D printing between layers and the optimal 3DP parameters chosen in this research.

**Figure 18:**
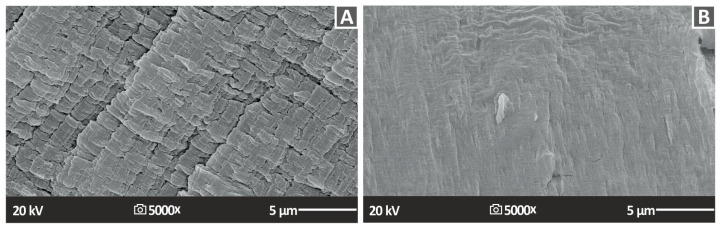
SEM photos of the morphology of the tensile samples’ fractured surface (**A**) PA12 2.0% ZrO_2_ 5000× fracture 5 μm, (**B**) PA12 4.0% ZrO_2_ 5000× fracture 5 μm.

**Figure 19:**
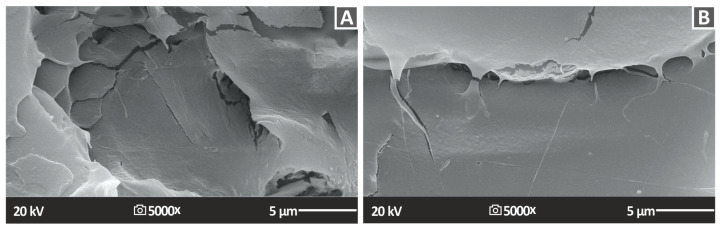
SEM photos of the morphology of the tensile samples’ fractured surface (**A**) PLA 2.0% ZrO_2_ 5000× fracture 5 μm, (**B**) PLA 4.0% ZrO_2_ 5000× fracture 5 μm.

Figure 18 and Figure 19 show the fractography analyses of the fractured surfaces of the tensile specimens for PA12 and PLA nanocompounds, respectively. In the case of PA12/ZrO_2_ nanocomposites, a reasonably ductile fracture mechanism was observed in all cases except for the PA12/ZrO_2_ (1.0 wt.%) specimen, which had a rough fractured surface and visible 3DP filaments. However, there were no visible filaments from different additively manufactured layers, indicating good interlayer fusion and optimal 3DP manufacturing parameters. For PLA/ZrO_2_ nanocomposites, a relatively brittle fracture mechanism was observed in all cases with typical brittle fracturing morphology. The fracture surfaces of the samples showed high quality, with no obvious intralayer voids except for certain voids in the PLA/ZrO_2_ (4.0 wt.%) sample, which indicated the optimal 3DP factors chosen for PLA and PLA/ZrO_2_ nanocompounds in this research [109,110].

## 4. Discussion

**Figure 20:**
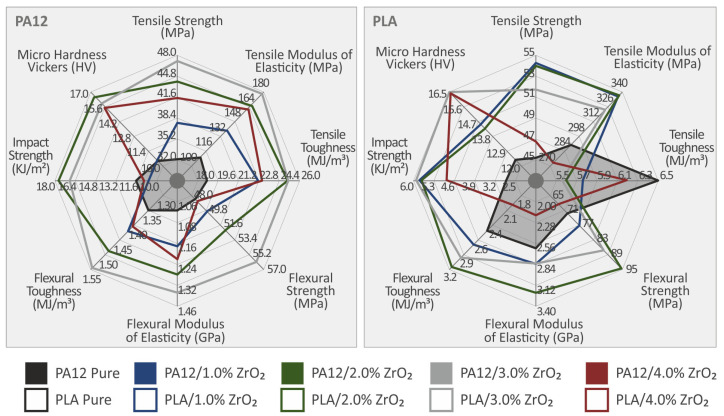
A graph in the shape of a spider web summarizes the mechanical characteristics of raw PA12 and PLA in comparison to their respective nanocomposites with ZrO_2_ filler loading at different concentrations (1.0, 2.0, 3.0, and 4.0 wt.%). The shaded area in the graph represents the mechanical performance of the raw materials. The legend provides information about the color corresponding to each material represented in the graph.

Herein, according to the authors’ best knowledge, in the literature, for the first time, zirconium dioxide was evaluated as a reinforcement agent in MEX 3D printing. Furthermore, its performance was evaluated against two popular polymeric matrices, i.e., PA12 and PLA, which are both related to medical applications. PA12 is a medical-grade polymer, while PLA is, by itself, a biocompatible polymer. Therefore, the idea was to use a material commonly used in medical applications, such as zirconium dioxide (zirconia), as reinforcement, and this was achieved; the produced nanocomposites may have a high potential for respective applications but also for other engineering applications. The use of ceramics as reinforcement in MEX 3D printing has shown potential, although the literature is still limited [111]. The hypothesis was proven, and zirconia managed to enhance the tensile strength of PA12 by 47.7% (with 3 wt.% loading) and the PLA by 20.1% (with 1.0 wt.% loading). So, the two matrices showed different reinforcement effects by the addition of zirconia, attributed probably to different interactions between the matrix and the filler. In the flexural tests, the reinforcing effect was in the same order as the tensile tests, but in this case, PLA showed higher improvement, with 31.1% (with 2.0 wt.% loading), than the PA12 polymer, which was improved compared to the unfilled matrix, by 16.0% (with 3.0 wt.% loading). The mechanical test results for the nanocompounds prepared with the two polymeric matrices are summarized in Figure 20. Overall, the 3 wt.% nanocomposite using PA12 as the matrix material and the 2 wt.% nanocomposite using PLA as the matrix material had the best performance in most of the conducted mechanical tests. The difference between their performance indicates that these results cannot be generalized for other polymeric matrices as well. Further experiments are required with each polymer to derive its improvement by the addition of the zirconia filler. Regarding the zirconia additive, loadings up to 4 wt.% were examined. This is because, on both polymeric matrices, at 4 wt.%, the mechanical performance started to decline, indicating that saturation of the zirconia on the nanocomposites started to occur. This negatively affects the mechanical performance of the nanocomposites [112,113].

For the fabrication of the nanocomposites, a thermomechanical process was followed that can be easily industrialized. In the higher magnification images of the fracture surfaces, no agglomerations of the zirconia filler could be located, and this was also confirmed with the EDS mapping in different regions of the surfaces. Additionally, the deviation in the mechanical tests was acceptable, indicating that the composition was similar in the nanocompounds in both matrices and all of the loadings evaluated. So, the NPs’ distribution in the matrix should have been well formed in the prepared samples. In the PLA polymer, it was not possible to acquire higher magnification images on SEM, as it was burned. PA12 allowed slightly higher magnification images on SEM, but nanocompounds were evaluated at the same magnification levels to be comparable. The addition of zirconia, as mentioned, did not affect the stability of the polymeric matrices under thermal loading, which is also a positive outcome for the prepared nanocomposites. Additionally, TGA showed that the nanocomposite start to degrade at higher temperatures than the ones used to process them in the current study. This is a positive result for the process followed and ensures that no such phenomena affected the acquired results.

The results presented herein cannot be directly evaluated with literature, as no similar nanocomposites for MEX 3D printing, prepared with the proposed methodology, have been presented so far. The addition of the titanium nitride ceramic in NP form in the PLA matrix, prepared with a similar process for the MEX 3D printing technique, achieved a slightly higher reinforcement effect [111] than the zirconia investigated herein. Still, the differences are not that high and the two ceramics are used for different types of applications. Non-ceramic additives in NP forms, such as alumina [47], prepared in a similar way for MEX 3D printing applications, achieved similar reinforcement effects on the two polymeric matrices.

## 5. Conclusions

The purpose of this research was to examine the effects of adding small quantities of zirconium dioxide (ZrO_2_) to 3D-printed nanocomposite filaments made of PA12 and PLA on their mechanical properties. The aim was to enhance the mechanical properties of the two popular polymeric matrices, which are often used in medical applications requiring medical-grade and biocompatible polymers, such as the two studied herein. Zirconium dioxide, which is also popular in the medical field, managed to increase the mechanical strength of the two polymers for all loadings considered in the work. Still, as reported, the response differed between the two polymers. The nanocomposites were produced by melt-mixing/compounding and intended for use as feedstocks in FFF 3D printing to enhance the mechanical properties of 3D-printed specimens.

In this study, PA12 and PLA were chosen as the polymeric materials, and different amounts (1.0, 2.0, 3.0, and 4.0 wt.%) of ZrO_2_ nanoparticles were added to them. The purpose was to examine the influence of ZrO_2_ nanocompounds on the mechanical characteristics of the 3DP nanocomposite filaments, which can be used in the medical field and various engineering applications, such as mechanical structures and machines as well as internal parts in the automotive, aerospace, and marine industries. The filaments were employed to create 3DP prototype specimens according to various ASTM protocols and then subjected to mechanical response tests, including tensile, flexural, impact, and microhardness investigations. SEM analyses of the side surface morphology and the tensile samples’ fractured surface morphology were also performed to evaluate the influence of nanofiller loading on the 3DP samples.

This study focused on examining the impact of zirconium dioxide (ZrO_2_) nanoparticles on the mechanical characteristics of Polyamide 12 and Polylactic Acid in 3D FFF printing. The research successfully determined the impact of ZrO_2_ nanocompounds on the mechanical responses of the materials. The future direction of this work will be to explore other properties, such as the electrical and optical properties, of these materials. The results of this study suggest that ZrO_2_ nanocomposites can be further investigated as fillers for developing multifunctional nanocomposites in 3D FFF printing.

## Figures and Tables

**Table 1:** Major Raman peaks of pure PLA identified and their corresponding determined assignments.

Wavenumber (cm^−1^)	Assignment of the Raman Peak
870	C-COO stretching [85]
1040	C-CH3 stretching [85]
1060	C-O-C stretching [86]
1126	C-O-C stretching [87]
1293	C-O-C stretching [87]; C-H_2_ twisting [88]
1413	C-H_3_ deformation [86]
1437	C-H_3_ deformation [86]; C-H_2_ deformation [88]
1457	C-H_3_ symmetric bending [85,86,87]; C-H_2_ twisting [88]
1770	C=O stretching [85,87]
2721	C=O stretching [89]
2845	C-H_2_ symmetric stretching [84]
2880	C-H_2_ symmetric stretching [84]; C-H symmetric stretching [90]
2945	C-H_2_ asymmetric stretching [84]
3000	C-H_3_ asymmetric stretch [90]

**Table 2:** Major Raman peaks of pure PA12 identified and their corresponding determined assignments.

Wavenumber (cm^−1^)	Assignment of the Raman Peak
1060	C-O-C stretching [86]
1105	C-O-C stretching [86]
1293	C-O-C stretching [86]
1434	C-H_2_ deformation [86,88]
2850	C-H_2_ symmetric stretching [84]
2884	C-H_2_ symmetric stretching [84]
2923	C-H_2_ asymmetric stretching [84]

## Data Availability

The data presented in this study are available upon request from the corresponding author.

## References

[1] Rayna T., Striukova L. (2016). From rapid prototyping to home fabrication: How 3D printing is changing business model innovation. Technol. Forecast. Soc. Chang..

[2] Ali H., Batai S., Sarbassov D. (2019). 3D printing: A critical review of current development and future prospects. Rapid Prototyp. J..

[3] Gupta M. (2017). 3D Printing of Metals. Metals.

[4] Valino A.D., Dizon J.R.C., Espera A.H., Chen Q., Messman J., Advincula R.C. (2019). Advances in 3D printing of thermoplastic polymer composites and nanocomposites. Prog. Polym. Sci..

[5] Chen Z., Li Z., Li J., Liu C., Lao C., Fu Y., Liu C., Li Y., Wang P., He Y. (2018). 3D printing of ceramics: A review. J. Eur. Ceram. Soc..

[6] Zhong S., Shi Q., Deng Y., Sun Y., Politis C., Yang S. (2022). High-performance zirconia ceramic additively manufactured via NanoParticle Jetting. Ceram. Int..

[7] Weißmann V., Drescher P., Bader R., Seitz H., Hansmann H., Laufer N. (2017). Comparison of Single Ti6Al4V Struts Made Using Selective Laser Melting and Electron Beam Melting Subject to Part Orientation. Metals.

[8] Herzberger J., Sirrine J.M., Williams C.B., Long T.E. (2019). Polymer Design for 3D Printing Elastomers: Recent Advances in Structure, Properties, and Printing. Prog. Polym. Sci..

[9] Wang B., Zhang Z., Pei Z., Qiu J., Wang S. (2020). Current progress on the 3D printing of thermosets. Adv. Compos. Hybrid Mater..

[10] Pervaiz S., Qureshi T.A., Kashwani G., Kannan S. (2021). 3D Printing of Fiber-Reinforced Plastic Composites Using Fused Deposition Modeling: A Status Review. Materials.

[11] Lopes L., Silva A., Carneiro O. (2018). Multi-material 3D printing: The relevance of materials affinity on the boundary interface performance. Addit. Manuf..

[12] Goh G.L., Zhang H., Chong T.H., Yeong W.Y. (2021). 3D Printing of Multilayered and Multimaterial Electronics: A Review. Adv. Electron. Mater..

[13] Ciubară A., Burlea Ș.L., Axinte M., Cimpoeșu R., Chicet D.L., Manole V., Burlea G., Cimpoeșu N. (2018). 3D Printer-Manufacturing of Complex Geometry Elements. IOP Conf. Ser. Mater. Sci. Eng..

[14] Scott P.J., Meenakshisundaram V., Hegde M., Kasprzak C.R., Winkler C.R., Feller K.D., Williams C.B., Long T.E. (2020). 3D Printing Latex: A Route to Complex Geometries of High Molecular Weight Polymers. ACS Appl. Mater. Interfaces.

[15] Maróti P., Kocsis B., Ferencz A., Nyitrai M., Lőrinczy D. (2020). Differential thermal analysis of the antibacterial effect of PLA-based materials planned for 3D printing. J. Therm. Anal. Calorim..

[16] Vidakis N., Petousis M., Mountakis N., Papadakis V., Moutsopoulou A. (2023). Mechanical strength predictability of full factorial, Taguchi, and Box Behnken designs: Optimization of thermal settings and Cellulose Nanofibers content in PA12 for MEX AM. J. Mech. Behav. Biomed. Mater..

[17] Vidakis N., Petousis M., Savvakis K., Maniadi A., Koudoumas E. (2019). A comprehensive investigation of the mechanical behavior and the dielectrics of pure polylactic acid (PLA) and PLA with graphene (GnP) in fused deposition modeling (FDM). Int. J. Plast. Technol..

[18] Vidakis N., Petousis M., Mountakis N., Korlos A., Papadakis V., Moutsopoulou A. (2022). Trilateral Multi-Functional Polyamide 12 Nanocomposites with Binary Inclusions for Medical Grade Material Extrusion 3D Printing: The Effect of Titanium Nitride in Mechanical Reinforcement and Copper/Cuprous Oxide as Antibacterial Agents. J. Funct. Biomater..

[19] Aati S., Shrestha B., Fawzy A. (2022). Cytotoxicity and antimicrobial efficiency of ZrO_2_ nanoparticles reinforced 3D printed resins. Dent. Mater..

[20] Vidakis N., Petousis M., Michailidis N., Mountakis N., Papadakis V., Argyros A., Charou C. (2023). Polyethylene glycol and polyvinylpyrrolidone reduction agents for medical grade polyamide 12/silver nanocomposites development for material extrusion 3D printing: Rheological, thermomechanical, and biocidal performance. React. Funct. Polym..

[21] Wei X., Jin M.-L., Yang H., Wang X.-X., Long Y.-Z., Chen Z. (2022). Advances in 3D printing of magnetic materials: Fabrication, properties, and their applications. J. Adv. Ceram..

[22] Alimi O.A., Akinnawo C.A., Meijboom R. (2020). Monolith catalyst design *via* 3D printing: A reusable support for modern palladium-catalyzed cross-coupling reactions. New J. Chem..

[23] Kumar P., Rajak D.K., Abubakar M., Ali S.G.M., Hussain M. (2021). 3D Printing Technology for Biomedical Practice: A Review. J. Mater. Eng. Perform..

[24] Triacca A., Pitzanti G., Mathew E., Conti B., Dorati R., Lamprou D.A. (2022). Stereolithography 3D printed implants: A preliminary investigation as potential local drug delivery systems to the ear. Int. J. Pharm..

[25] Do A.-V., Khorsand B., Geary S.M., Salem A.K. (2015). 3D Printing of Scaffolds for Tissue Regeneration Applications. Adv. Healthc. Mater..

[26] Shakibania S., Khakbiz M., Bektas C.K., Ghazanfari L., Banizi M.T., Lee K.-B. (2022). A review of 3D printing technology for rapid medical diagnostic tools. Mol. Syst. Des. Eng..

[27] Shapira A., Dvir T. (2021). 3D Tissue and Organ Printing—Hope and Reality. Adv. Sci..

[28] Chen A.Y., Pegg E., Chen A., Jin Z., Gu G.X. (2021). 4D Printing of Electroactive Materials. Adv. Intell. Syst..

[29] Shao L.-H., Zhao B., Zhang Q., Xing Y., Zhang K. (2020). 4D printing composite with electrically controlled local deformation. Extrem. Mech. Lett..

[30] Kodama H. (1981). Automatic method for fabricating a three-dimensional plastic model with photo-hardening polymer. Rev. Sci. Instrum..

[31] Vidakis N., Petousis M., Velidakis E., Mountakis N., Tzounis L., Liebscher M., Grammatikos S.A. (2021). Enhanced Mechanical, Thermal and Antimicrobial Properties of Additively Manufactured Polylactic Acid with Optimized Nano Silica Content. Nanomaterials.

[32] Shahrubudin N., Lee T., Ramlan R. (2019). An Overview on 3D Printing Technology: Technological, Materials, and Applications. Procedia Manuf..

[33] Vidakis N., Petousis M., Vairis A., Savvakis K., Maniadi A. (2017). On the compressive behavior of an FDM Steward Platform part. J. Comput. Des. Eng..

[34] Hossain I., Chowdhury M.A., Zahid S., Sakib-Uz-Zaman C., Rahaman M.L., Kowser A. (2021). Development and analysis of nanoparticle infused plastic products manufactured by machine learning guided 3D printer. Polym. Test..

[35] Aati S., Akram Z., Ngo H., Fawzy A.S. (2021). Development of 3D printed resin reinforced with modified ZrO_2_ nanoparticles for long-term provisional dental restorations. Dent. Mater..

[36] García E., Núñez P., Chacón J., Caminero M., Kamarthi S. (2020). Comparative study of geometric properties of unreinforced PLA and PLA-Graphene composite materials applied to additive manufacturing using FFF technology. Polym. Test..

[37] Petousis M., Michailidis N., Papadakis V.M., Korlos A., Mountakis N., Argyros A., Dimitriou E., Charou C., Moutsopoulou A., Vidakis N. (2023). Optimizing the Rheological and Thermomechanical Response of Acrylonitrile Butadiene Styrene/Silicon Nitride Nanocomposites in Material Extrusion Additive Manufacturing. Nanomaterials.

[38] Vidakis N., Petousis M., Mountakis N., Papadakis V., Charou C., Rousos V., Bastas P. (2023). Glass Fillers in Three Different Forms Used as Reinforcement Agents of Polylactic Acid in Material Extrusion Additive Manufacturing. Appl. Sci..

[39] Farhan Han S.N.M., Mastura M.T., Mansor M.R., Sapuan S.M., Mansor M.R. (2021). Chapter 14—Recent progress on fused filament fabrication research: Sustainable materials and processing parameters. Design for Sustainability.

[40] Vidakis N., Petousis M., Velidakis E., Korlos A., Kechagias J.D., Tsikritzis D., Mountakis N. (2022). Medical-Grade Polyamide 12 Nanocomposite Materials for Enhanced Mechanical and Antibacterial Performance in 3D Printing Applications. Polymers.

[41] Petousis M., Vidakis N., Mountakis N., Karapidakis E., Moutsopoulou A. (2023). Functionality Versus Sustainability for PLA in MEX 3D Printing: The Impact of Generic Process Control Factors on Flexural Response and Energy Efficiency. Polymers.

[42] El Magri A., Vaudreuil S. (2021). Optimizing the mechanical properties of 3D-printed PLA-graphene composite using response surface methodology. Arch. Mater. Sci. Eng..

[43] Cojocaru V., Frunzaverde D., Miclosina C.-O., Marginean G. (2022). The Influence of the Process Parameters on the Mechanical Properties of PLA Specimens Produced by Fused Filament Fabrication—A Review. Polymers.

[44] Kechagias J.D., Vidakis N., Petousis M., Mountakis N. (2022). A multi-parametric process evaluation of the mechanical response of PLA in FFF 3D printing. Mater. Manuf. Process..

[45] Petousis M., Vidakis N., Mountakis N., Papadakis V., Kanellopoulou S., Gaganatsiou A., Stefanoudakis N., Kechagias J. (2022). Multifunctional Material Extrusion 3D-Printed Antibacterial Polylactic Acid (PLA) with Binary Inclusions: The Effect of Cuprous Oxide and Cellulose Nanofibers. Fibers.

[46] Petousis M., Ninikas K., Vidakis N., Mountakis N., Kechagias J.D. (2023). Multifunctional PLA/CNTs nanocomposites hybrid 3D printing integrating material extrusion and CO_2_ laser cutting. J. Manuf. Process..

[47] Petousis M., Vidakis N., Mountakis N., Papadakis V., Tzounis L. (2022). Three-Dimensional Printed Polyamide 12 (PA12) and Polylactic Acid (PLA) Alumina (Al_2_O*_3_*) Nanocomposites with Significantly Enhanced Tensile, Flexural, and Impact Properties. Nanomaterials.

[48] Kechagias J.D., Vidakis N., Ninikas K., Petousis M., Vaxevanidis N.M. (2022). Hybrid 3D printing of multifunctional polylactic acid/carbon black nanocomposites made with material extrusion and post-processed with CO_2_ laser cutting. Int. J. Adv. Manuf. Technol..

[49] Vidakis N., David C., Petousis M., Sagris D., Mountakis N., Moutsopoulou A. (2022). The effect of six key process control parameters on the surface roughness, dimensional accuracy, and porosity in material extrusion 3D printing of polylactic acid: Prediction models and optimization supported by robust design analysis. Adv. Ind. Manuf. Eng..

[50] Vidakis N., Petousis M., Kechagias J. (2022). Parameter effects and process modelling of Polyamide 12 3D-printed parts strength and toughness. Mater. Manuf. Process..

[51] Vidakis N., Petousis M., Velidakis E., Mountakis N., Grammatikos S., Tzounis L. (2023). Multi-functional medical grade Polyamide12/Carbon black nanocomposites in material extrusion 3D printing. Compos. Struct..

[52] Pejkowski Ł., Seyda J., Nowicki K., Mrozik D. (2023). Mechanical performance of non-reinforced, carbon fiber reinforced and glass bubbles reinforced 3D printed PA12 polyamide. Polym. Test..

[53] Razaviye M.K., Tafti R.A., Khajehmohammadi M. (2022). An investigation on mechanical properties of PA12 parts produced by a SLS 3D printer: An experimental approach. CIRP J. Manuf. Sci. Technol..

[54] Kechagias J.D., Vidakis N. (2022). Parametric optimization of material extrusion 3D printing process: An assessment of Box-Behnken vs. full-factorial experimental approach. Int. J. Adv. Manuf. Technol..

[55] Zhou L., Fu J., He Y. (2020). A Review of 3D Printing Technologies for Soft Polymer Materials. Adv. Funct. Mater..

[56] Clarissa W.H.-Y., Chia C.H., Zakaria S., Evyan Y.C.-Y. (2021). Recent advancement in 3-D printing: Nanocomposites with added functionality. Prog. Addit. Manuf..

[57] Tran T.Q., Ng F.L., Kai J.T.Y., Feih S., Nai M.L.S. (2022). Tensile Strength Enhancement of Fused Filament Fabrication Printed Parts: A Review of Process Improvement Approaches and Respective Impact. Addit. Manuf..

[58] Gensler R., Gröppel P., Muhrer V., Müller N. (2002). Application of Nanoparticles in Polymers for Electronics and Electrical Engineering. Part. Part. Syst. Charact..

[59] Khan I., Saeed K., Khan I. (2019). Nanoparticles: Properties, applications and toxicities. Arab. J. Chem..

[60] Maťátková O., Michailidu J., Miškovská A., Kolouchová I., Masák J., Čejková A. (2022). Antimicrobial properties and applications of metal nanoparticles biosynthesized by green methods. Biotechnol. Adv..

[61] Dash K.K., Deka P., Bangar S.P., Chaudhary V., Trif M., Rusu A. (2022). Applications of Inorganic Nanoparticles in Food Packaging: A Comprehensive Review. Polymers.

[62] Kumar S., Singh R., Singh T., Batish A. (2023). Fused filament fabrication: A comprehensive review. J. Thermoplast. Compos. Mater..

[63] Kam M., Ipekçi A., Şengül Ö. (2021). Investigation of the effect of FDM process parameters on mechanical properties of 3D printed PA12 samples using Taguchi method. J. Thermoplast. Compos. Mater..

[64] Moskalyuk O.A., Belashov A.V., Zhikhoreva A.A., Beltukov Y.M., Semenova I.V. (2023). Mechanical Performance of Polystyrene-Based Nanocomposites Filled with Carbon Allotropes. Appl. Sci..

[65] Patel A., Taufik M. (2021). Nanocomposite materials for fused filament fabrication. Mater. Today Proc..

[66] Hu C., Sun J., Long C., Wu L., Zhou C., Zhang X. (2019). Synthesis of nano zirconium oxide and its application in dentistry. Nanotechnol. Rev..

[67] Sollazzo V., Pezzetti F., Scarano A., Piattelli A., Bignozzi C.A., Massari L., Brunelli G., Carinci F. (2008). Zirconium oxide coating improves implant osseointegration in vivo. Dent. Mater..

[68] Kumari L., Li W.Z., Xu J.M., Leblanc R.M., Wang D.Z., Li Y., Guo H., Zhang J. (2009). Controlled Hydrothermal Synthesis of Zirconium Oxide Nanostructures and Their Optical Properties. Cryst. Growth Des..

[69] Schweiger J., Bomze D., Schwentenwein M. (2019). 3D Printing of Zirconia–What is the Future?. Curr. Oral Health Rep..

[70] Zhu Y., Liu K., Deng J., Ye J., Ai F., Ouyang H., Wu T., Jia J., Cheng X., Wang X. (2019). 3D printed zirconia ceramic hip joint with precise structure and broad-spectrum antibacterial properties. Int. J. Nanomed..

[71] Shannon A., O’sullivan K.J., Clifford S., O’sullivan L. (2022). Assessment and selection of filler compounds for radiopaque PolyJet multi-material 3D printing for use in clinical settings. Proc. Inst. Mech. Eng. Part H J. Eng. Med..

[72] Sokolov P.S., Komissarenko D.A., Dosovitskii G.A., Shmeleva I.A., Slyusar I.V., Dosovitskii A.E. (2018). Rheological Properties of Zirconium Oxide Suspensions in Acrylate Monomers for Use In 3D Printing. Glas. Ceram..

[73] Sokolov P.S., Komissarenko D.A., Shmeleva I.A., Slyusar I.V., Dosovitskiy G.A., Evdokimov P.V., Putlyaev V.I., Dosovitskiy A.E. (2018). Suspensions on the Basis of Stabilised Zirconium Oxide for Three-Dimensional Printing. IOP Conf. Ser. Mater. Sci. Eng..

[74] Rahim T.N.A.T., Abdullah A.M., Akil H., Mohamad D. (2016). Comparison of mechanical properties for polyamide 12 composite-based biomaterials fabricated by fused filament fabrication and injection molding. AIP Conf. Proc..

[75] Clemens F., Schulz J., Gorjan L., Liersch A., Sebastian T., Sarraf F., Meboldt M., Klahn C. (2021). Debinding and Sintering of Dense Ceramic Structures Made with Fused Deposition Modeling BT—Industrializing Additive Manufacturing.

[76] Gad M.M., Al-Harbi F.A., Akhtar S., Fouda S.M. (2022). 3D-Printable Denture Base Resin Containing SiO_2_ Nanoparticles: An In Vitro Analysis of Mechanical and Surface Properties. J. Prosthodont..

[77] Lyu Y., Wen X., Wang G., Zhang Q., Lin L., Schlarb A.K., Shi X. (2022). 3D printing nanocomposites with controllable “strength-toughness” transition: Modification of SiO_2_ and construction of Stereocomplex Crystallites. Compos. Sci. Technol..

[78] Jeong G., Park C.H., Kim B.-Y., Kim J., Park S.-D., Yang H., Lee W.S. (2020). Photocurable Elastomer Composites with SiO_2_-Mediated Cross-Links for Mechanically Durable 3D Printing Materials. ACS Appl. Polym. Mater..

[79] Cleetus C.M., Primo F.A., Fregoso G., Raveendran N.L., Noveron J.C., Spencer C.T., Ramana C.V., Joddar B. (2020). Alginate Hydrogels with Embedded ZnO Nanoparticles for Wound Healing Therapy. Int. J. Nanomed..

[80] Tan H.W., An J., Chua C.K., Tran T. (2019). Metallic Nanoparticle Inks for 3D Printing of Electronics. Adv. Electron. Mater..

[81] Bennett C., Sojithamporn P., Thanakulwattana W., Wattanutchariya W., Leksakul K., Nakkiew W., Jantanasakulwong K., Rachtanapun P., Suhr J., Sawangrat C. (2022). Optimization of 3D Printing Technology for Fabrication of Dental Crown Prototype Using Plastic Powder and Zirconia Materials. Materials.

[82] Wang Q., Ma Z., Wang Y., Zhong L., Xie W. (2020). Fabrication and characterization of 3D printed biocomposite scaffolds based on PCL and zirconia nanoparticles. Bio-Des. Manuf..

[83] Khattar A., Alsaif M.H., Alghafli J.A., Alshaikh A.A., Alsalem A.M., Almindil I.A., Alsalman A.M., Alboori A.J., Al-Ajwad A.M., Almuhanna H.M. (2022). Influence of ZrO_2_ Nanoparticle Addition on the Optical Properties of Denture Base Materials Fabricated Using Additive Technologies. Nanomaterials.

[84] Makarem M., Lee C.M., Kafle K., Huang S., Chae I., Yang H., Kubicki J.D., Kim S.H. (2019). Probing cellulose structures with vibrational spectroscopy. Cellulose.

[85] Lin Z., Guo X., He Z., Liang X., Wang M., Jin G. (2020). Thermal degradation kinetics study of molten polylactide based on Raman spectroscopy. Polym. Eng. Sci..

[86] Stuart B.H. (1996). Temperature studies of polycarbonate using Fourier transform Raman spectroscopy. Polym. Bull..

[87] Resta V., Quarta G., Lomascolo M., Maruccio L., Calcagnile L. (2015). Raman and Photoluminescence spectroscopy of polycarbonate matrices irradiated with different energy 28Si+ ions. Vacuum.

[88] Zimmerer C., Matulaitiene I., Niaura G., Reuter U., Janke A., Boldt R., Sablinskas V., Steiner G. (2018). Nondestructive characterization of the polycarbonate-octadecylamine interface by surface enhanced Raman spectroscopy. Polym. Test..

[89] Zou H., Yi C., Wang L., Liu H., Xu W. (2009). Thermal degradation of poly(lactic acid) measured by thermogravimetry coupled to Fourier transform infrared spectroscopy. J. Therm. Anal. Calorim..

[90] Liu X., Zou Y., Li W., Cao G., Chen W. (2006). Kinetics of thermo-oxidative and thermal degradation of poly(d,l-lactide) (PDLLA) at processing temperature. Polym. Degrad. Stab..

[91] Akhoundi B., Hajami F. (2022). Extruded polymer instability study of the polylactic acid in fused filament fabrication process: Printing speed effects on tensile strength. Polym. Eng. Sci..

[92] Zhang Z., Gkartzou E., Jestin S., Semitekolos D., Pappas P.-N., Li X., Karatza A., Zouboulis P., Trompeta A.-F., Koutroumanis N. (2022). 3D Printing Processability of a Thermally Conductive Compound Based on Carbon Nanofiller-Modified Thermoplastic Polyamide 12. Polymers.

[93] García E., Núñez P., Caminero M., Chacón J., Kamarthi S. (2022). Effects of carbon fibre reinforcement on the geometric properties of PETG-based filament using FFF additive manufacturing. Compos. Part B Eng..

[94] Golbang A., Harkin-Jones E., Wegrzyn M., Campbell G., Archer E., McIlhagger A. (2019). Production and characterization of PEEK/IF-WS2 nanocomposites for additive manufacturing: Simultaneous improvement in processing characteristics and material properties. Addit. Manuf..

[95] Ferreira I., Machado M., Alves F., Marques A.T. (2019). A review on fibre reinforced composite printing via FFF. Rapid Prototyp. J..

[96] Sevastaki M., Suchea M.P., Kenanakis G. (2020). 3D Printed Fully Recycled TiO_2_-Polystyrene Nanocomposite Photocatalysts for Use against Drug Residues. Nanomaterials.

[97] Jo H.Y., Jung D.S., Lee S.-H., Kim D.S., Lee Y.K., Lim H.M. (2016). Characterization of Composites Prepared with Polyamide-Imide and Alumina Synthesized by Solvothermal Method. Nanosci. Nanotechnol. Lett..

[98] Nakonieczny D.S., Kern F., Dufner L., Antonowicz M., Matus K. (2021). Alumina and Zirconia-Reinforced Polyamide PA-12 Composites for Biomedical Additive Manufacturing. Materials.

[99] Nakonieczny D.S., Antonowicz M., SimhaMartynkova G., Kern F., Pazourková L., Erfurt K., Hüpsch M. (2022). PA-12-Zirconia-Alumina-Cenospheres 3D Printed Composites: Accelerated Ageing and Role of the Sterilisation Process for Physicochemical Properties. Polymers.

[100] Qi S., Gao X., Su Y., Zhou Y., Dong X., Wang D. (2022). Effect of carbon nanotubes on mechanical properties of polyamide 12 parts by fused filament fabrication. Polymer.

[101] Klouda K., Kubátová H., Nechvátal M., Bátrlová K., Roupcová P. (2021). Measurement of nanoparticles in 3d printing using fff/fdm technology. Proceedings of the NANOCON Conference Proceedings—International Conference on Nanomaterials.

[102] Li C., Liu Y., Chen Z. (2023). Study of Mechanical Properties of Micron Polystyrene-Toughened Epoxy Resin. Appl. Sci..

[103] Bouamer A., Younes A. (2022). Effect of ZnO, SiO_2_ and Al_2_O_3_ Doped on Morphological, Optical, Structural and Mechanical Properties of Polylactic Acid. Key Eng. Mater..

[104] Khattar A., Alghafli J.A., Muheef M.A., Alsalem A.M., Al-Dubays M.A., AlHussain H.M., AlShoalah H.M., Khan S.Q., AlEraky D.M., Gad M.M. (2023). Antibiofilm Activity of 3D-Printed Nanocomposite Resin: Impact of ZrO_2_ Nanoparticles. Nanomaterials.

[105] Stojšić J., Raos P., Milinović A., Damjanović D. (2022). A Study of the Flexural Properties of PA12/Clay Nanocomposites. Polymers.

[106] Wang Y., Shi J., Liu Z. (2020). Bending performance enhancement by nanoparticles for FFF 3D printed nylon and nylon/Kevlar composites. J. Compos. Mater..

[107] Vidakis N., Petousis M., Vairis A., Savvakis K., Maniadi A. (2019). A parametric determination of bending and Charpy’s impact strength of ABS and ABS-plus fused deposition modeling specimens. Prog. Addit. Manuf..

[108] Afshari M., Bakhshi S., Samadi M.R., Afshari H. (2022). Optimizing the mechanical properties of TiO_2_/PA12 nano-composites fabricated by SLS 3D printing. Polym. Eng. Sci..

[109] Xu W., Jambhulkar S., Zhu Y., Ravichandran D., Kakarla M., Vernon B., Lott D.G., Cornella J.L., Shefi O., Miquelard-Garnier G. (2021). 3D printing for polymer/particle-based processing: A review. Compos. Part B Eng..

[110] He F., Khan M. (2021). Effects of Printing Parameters on the Fatigue Behaviour of 3D-Printed ABS under Dynamic Thermo-Mechanical Loads. Polymers.

[111] Petousis M., Vidakis N., Mountakis N., Moutsopoulou A., Papadakis V., Maravelakis E. (2023). On the substantial mechanical reinforcement of Polylactic Acid with Titanium Nitride ceramic nanofillers in material extrusion 3D printing. Ceram. Int..

[112] Bhattacharya M., Bhowmick A.K. (2010). Synergy in carbon black-filled natural rubber nanocomposites. Part I: Mechanical, dynamic mechanical properties, and morphology. J. Mater. Sci..

[113] Zaragoza J., Fukuoka S., Kraus M., Thomin J., Asuri P. (2018). Exploring the Role of Nanoparticles in Enhancing Mechanical Properties of Hydrogel Nanocomposites. Nanomaterials.

